# Predicting Lymph Node Metastases in Patients with Biopsy-Proven Ductal Carcinoma In Situ of the Breast: Development and Validation of the DCIS-met Model

**DOI:** 10.1245/s10434-022-12900-7

**Published:** 2022-12-10

**Authors:** Claudia J. C. Meurs, Joost van Rosmalen, Marian B. E. Menke-Pluijmers, Sabine Siesling, Pieter J. Westenend

**Affiliations:** 1 Department of Health Technology and Services Research, Technical Medical Centre, University of Twente, Enschede, The Netherlands; 2 CMAnalyzing, Zevenaar, The Netherlands; 3 Department of Biostatistics, Erasmus MC, Rotterdam, The Netherlands; 4 Department of Epidemiology, Erasmus MC, Rotterdam, The Netherlands; 5 Department of Surgery, Albert Schweitzer Hospital, Dordrecht, The Netherlands; 6 Department of Research, Netherlands Comprehensive Cancer Organisation, Utrecht, The Netherlands; 7 Laboratory of Pathology Dordrecht, Dordrecht, The Netherlands

## Abstract

**Purpose:**

In patients with a biopsy-proven ductal carcinoma in situ (DCIS), axillary staging is frequently performed, but in hindsight often turns out to be superfluous. The aim of this observational study was to develop a prediction model for risk of lymph node metastasis in patients with a biopsy-proven DCIS.

**Methods:**

Data were received from the Dutch Pathology Databank and the Netherlands Cancer Registry. The population-based cohort consisted of all biopsy-proven DCIS patients diagnosed in the Netherlands in 2011 and 2012. The prediction model was evaluated with the area under the curve (AUC) of the receiver operating characteristic, and a calibration plot and a decision curve analysis and was validated in a Dutch cohort of patients diagnosed in the period 2016–2019.

**Results:**

Of 2892 biopsy-proven DCIS patients, 127 had metastasis (4.4%). Risk factors were younger age (OR = 0.97, 95% CI 0.95–0.99), DCIS not detected by screening (OR = 1.55, 95% CI 1.01–2.38), suspected invasive component at biopsy (OR = 1.86, 95% CI 1.01–3.41), palpable tumour (OR = 2.06, 95% CI 1.34–3.18), BI-RADS score 5 (OR = 2.41, 95% CI 1.53–3.78), intermediate-grade DCIS (OR = 3.01, 95% CI 1.27–7.15) and high-grade DCIS (OR = 3.20, 95% CI 1.36–7.54). For 24% (*n* = 708) of the patients, the predicted risk of lymph node metastasis was above 5%. Based on the decision curve analysis, the model had a net benefit for a predicted risk below 25%. The AUC was 0.745. Of the 2269 patients in the validation cohort, 53 (2.2%) had metastasis and the AUC was 0.741.

**Conclusions:**

This DCIS-met model can support clinical decisions on axillary staging in patients with biopsy-proven DCIS.

**Supplementary Information:**

The online version contains supplementary material available at 10.1245/s10434-022-12900-7.

It is still unclear when patients with ductal carcinoma in situ (DCIS) on biopsy should undergo axillary staging, which currently is mostly a sentinel lymph node biopsy (SLNB). SLNB is not considered necessary if the diagnosis at excision remains DCIS, because DCIS is non-invasive and will not metastasize, and lymph node (LN) metastases in these patients are rare.^1,2^ Nevertheless, SLNB is often performed for patients with a DCIS diagnosis at biopsy^3,4^ because 20% of the patients with a preoperative DCIS diagnosis are upstaged to invasive breast cancer at excision.^5^ In a previous study, we developed a model to predict the risk of upstaging to invasive breast cancer (https://www.evidencio.com/models/show/1074). Although we assume that patients at high risk for upstaging are also at risk for LN metastasis, the model for upstaging is merely an indirect assessment of the risk of axillary LN metastasis.

Currently, SLNB is offered to patients depending on the presence of risk factors for upstaging to invasive carcinoma at surgery.^6,7^ In hindsight, SLNB often turns out to be superfluous, and support is needed for physicians and patients in deciding whether or not an SLNB should be performed.

A large study by Francis et al. based on 1234 patients showed in univariable analysis that preoperative risk factors for LN metastasis were a palpable tumour, the papillary subtype of DCIS, and suspicion of microinvasion. In multivariable analysis, only the papillary subtype could be identified as a preoperative risk factor. A study by Lai et al. on 682 patients found an association of the mammographic size and the risk of metastases.^8^ Uemoto et al. showed that size predicts metastases with an AUC of 0.67.^9^ A study by Kim et al.^10^ on 406 patients found that size > 20 mm was associated with a higher risk of metastasis, and the authors developed a prediction model with an AUC of 0.75, including the variables size, palpability and grade.

However, it is not clear whether the patients included by Kim et al.^5^ are comparable with the Dutch population, since the upstaging rate in their study was 46%, which is more than double the upstaging rate in a Dutch cohort. Also, the model of Kim et al.^10^ was not validated and was based on only 20 events, hence that study has limitations. Studies on predicting LN metastases based on preoperatively known risk factors are still limited in number, but these studies are needed.

The aim of the present study was to develop a prediction model for axillary LN metastasis with DCIS on biopsy based on a nationwide cohort and to validate the developed model in a second cohort.

## Methods

This study was based on retrospective cohorts of patients with biopsy-proven DCIS diagnosed in the Netherlands. Data from both cohorts were registered in the Dutch Pathology Registry (PALGA) and the Netherlands Cancer Registry (NCR). PALGA is the Dutch Nationwide Pathology Databank.^11^ The NCR is hosted by the Netherlands Comprehensive Cancer Organisation and registers additional preoperative data and information on metastases. These two standard registries do not collect data on race or ethnicity. The data from the development cohort were nationwide and consisted of cases with incidence dates from January 2011 to June 2012. The process of selecting biopsy-proven DCIS and coding data was described in more detail in our previous study.^5^ The data for the validation cohort were selected from pathology reports that were recorded in a standardized structured reporting module of the PALGA. Patients were included if the incidence date was between July 2016 and March 2019 and if no information was missing that was needed to validate the model. To assess whether LN metastasis was missing for the patients who did not undergo axillary staging, we checked PALGA for metastases diagnosed at a later moment in time. Therefore, all cases with an excision diagnosis of invasive breast cancer were checked for LN metastasis in the PALGA registry; for the development cohort, we checked until April 2014, and for the validation cohort, until February 2021. For patients with a DCIS diagnosis after surgery, we partially checked the post-operative PALGA records for metastases. Axillary ultrasound examination is routinely performed, and enlarged lymph nodes are biopsied with fine needle aspiration. Patients with positive needle aspirations were excluded.

### Statistical Methods

The clinical workflow is presented in a chart. The distribution of potential risk factors was compared between cases with and without LN metastasis, using the Mann–Whitney *U* test, the Pearson chi-square test, and Fisher’s exact test.

A prediction model was developed based on multivariable logistic regression analysis. Since we expected that the risk factors we previously included in the risk model for upstaging to invasive breast cancer would also be risk factors for metastasis, we tested the following variables: detection mode, palpability, BI-RADS score, DCIS grade and suspected invasive component at biopsy.^5^ In addition, age was added to the model and tested as a continuous variable using both linear and quadratic terms [using the transformation (age − mean age)^2^] and using restricted cubic spline functions. Interaction effects were tested for combinations of independent variables that were clinically the most plausible: DCIS grade and age. Two-way interactions effects with *p* < 0.05 were included in the final model. Missing data in the potential risk factors were accounted for by means of multiple imputation with fully conditional specification. Twenty data sets with imputed data for the missing data were generated, and the results were pooled according to Rubin’s rules.

The goodness-of-fit of the developed model was analysed using the area under the curve (AUC) of the receiver operating characteristic (ROC) curve and a calibration plot. For internal validation of the model, bootstrap replications of the logistic regression were performed (100 times). The developed model was applied to the data of the validation cohort, which only included patients without missing data. For the validation cohort, a calibration plot was made as well, as was the ROC curve. For the ROC curve, the AUC was calculated, as was the maximum value of Youden’s index.

The risk of an LN metastasis was calculated for each patient of the development cohort, and the distribution of predicted risks was analysed. The clinical utility of the model was analysed on that cohort using decision curve analysis.^12^ In that analysis, the net benefit is calculated as true positives minus false positives multiplied by the number of false positives that are worth one true positive, divided by the total number of patients. The odds of the risk thresholds represent the number of SLNBs that one is willing to perform unnecessarily to find one metastasis. A low-risk threshold is chosen when one is worried about patients having metastasis and a high-risk threshold is chosen when one is worried about performing SLNB. The net benefit is calculated for a range of risk thresholds and presented graphically. In the decision curve analysis, the net benefit is also calculated when all patients are selected, as is the net benefit when no patient is selected. For a model to be clinically useful, the net benefit of the model needs to be higher than the net benefit when all or no patients are selected.

The model was developed in R. The following packages were used: the mice package for multiple imputation, the rms package for the evaluation of the predictive performance, and the rmda package for decision curve analysis. All other analyses were performed with STATA statistics/data analysis, version 13.1, StataCorp, Texas. All statistical tests were two-sided with a significance level of 0.05.

## Results

### Development Cohort

The study included 2892 patients in the model development cohort. The development cohort comprised 127 patients with LN metastases (4.4% of 2892 patients), which were found at first or secondary axillary evaluation (see Supplement 1). In 1899 (66%) of the patients, an SLNB was performed before or at the first excision. At first resection, 1821 patients underwent breast-conserving surgery and 1071 patients underwent mastectomy. The average SLNB rate of 66% consisted of 53% for breast-conserving surgery (*n* = 962) and 87% for mastectomy (*n* = 937). Of the 1899 patients that underwent SLNB, 95 (4.9%) had one or more positive lymph nodes (2.1% micro-metastasis, 2.7% macro-metastasis, 0.2% metastasis but size unknown). In addition, 2.7% of the 1899 cases had isolated tumour cells (ITC) [which we consider N0(i+)].

Of the 127 patients with metastasis, 113 were diagnosed with invasive breast cancer (for characteristics see Supplement 2). The remaining 14 patients were diagnosed with DCIS at excision; one had metastasis of unknown size, six had micro-metastasis and seven had macro-metastasis.

Of a total of 603 patients with invasive breast cancer after excision, 235 (39%) had an indication for adjuvant chemotherapy. For 122 of these patients, this indication was based only on tumour size or on the combination of tumour size and Her2Neu receptor status, grade or age; for 71 patients, the indication was based both on tumour characteristics and on positive lymph nodes, and for 42 patients, it was based only on positive lymph nodes. These 42 patients represented 1.5% of all the patients included in the model development cohort, 7% of all the included patients with invasive breast cancer and 37% of all the included patients with invasive breast cancer as well as LN metastasis.

### Validation Cohort

In the model validation cohort, 2269 patients were included. Of these 2269 patients, 49 (2.2%) had metastases that were found at primary or secondary axillary evaluation, including 24 patients with micro-metastases and 25 patients with macro-metastases. Besides these 49 patients, five patients had LN metastasis, which were found 18–45 months after surgery, together with invasive breast cancer. These five patients were all diagnosed with pure DCIS at excision, and SNLB was performed for two of these patients with no metastasis at that time. The rate of upstaging to invasive carcinoma at surgery was 17.4%. In 7 patients with LN metastases, the diagnosis was not upstaged at surgery. Details of patients with LN metastases are shown in Supplement 3; the risk of metastases was 2.3% for patients with intermediate-grade DICIS, 2.6% for patients with high-grade DCIS, 3.8% for patients with DCIS detected outside the screening, 5.9% for patients with a palpable tumour, 7.5% for patients with BI-RADS score 5, and 10.4% for patients with a suspected invasive component at biopsy.

### Model Development

The number of events in the development cohort (i.e., 127 patients with LN metastases) was sufficient to develop a model with six variables, since at least 10 events are needed per variable in a prediction model. The checks in the pathology records on metastases did not reveal any LN metastases and therefore cases without axillary staging (pN unknown) were considered N0.

Table 1 shows patient and biopsy characteristics and their relationship with LN metastasis (average 4.4%). LN metastases were found in 38 (2.1%) patients that underwent breast-conserving surgery and in 89 (8.3%) patients that underwent mastectomy. If there was a suspicion of an invasive component at biopsy, 10.1% had LN metastasis, whereas the metastasis rate was 4.1% if there was no suspicion. For palpable biopsy-proven DCIS, the metastasis rate was 10.1%, whereas it was 4.1% if the DCIS was not palpable. For biopsy-proven DCIS with BI-RADS score 5, the LN metastasis rate was 11.8%, versus 3.5% for BI-RADS score 4 and 2.8% for BI-RADS score 3. Table 2 shows the results of the multivariable analyses of the risk for lymph node metastasis (*n* = 127) based on the six preoperatively known potential risk factors.

**Table 1:** Distribution of lymph node metastases in relation to preoperatively known features in the model development cohort

	Biopsies with DCIS	Lymph node metastasis				
	*N*	No		Yes		*p*-value
		*N*	(%)	*N*	(%)	
Total	2892	2765	95.6	127	4.4	
Age, years						
Mean (range)	58.7 (24-91)	58.9 (24–91)		53.9 (26-82)		< 0.001
Detection mode						< 0.001
Screen-detected	1850	1797	97.1	53	2.9	
Otherwise	961	891	92.7	70	7.3	
Missing	81	77	95.1	4	4.9	
Palpable						< 0.001
No	2147	2086	97.2	61	2.8	
Yes	597	538	90.1	59	9.9	
Missing	148	141	95.3	7	4.7	
BI-RADS score						< 0.001
3	365	355	97.3	10	2.7	
4	1996	1926	96.5	70	3.5	
5	308	272	88.3	36	11.7	
Missing	223	212	95.1	11	4.9	
DCIS histological grade at biopsy						0.003
Low	422	416	98.6	6	1.4	
Intermediate	1083	1036	95.7	47	4.3	
High	1303	1234	94.7	69	5.3	
Missing	84	79	94.0	5	6.0	
Suspected invasive component at biopsy						0.001
No	2743	2631	95.6	112	4.1	
Yes	149	134	89.9	15	10.1	
Synchronous contralateral breast tumour						0.366
No	2796	2675	95.7	121	4.3	
Yes	96	90	93.8	6	6.3	
Preoperative MRI						0.006
No (or unknown)	2188	2105	96.2	83	3.8	
Yes	704	660	94.8	44	6.3	
Preoperative multidisciplinary team meeting						0.339
No (or unknown)	301	291	96.7	10	3.3	
Yes	2591	2474	95.5	117	4.5	
1st resection						< 0.001
Breast-conserving surgery	1821	1783	97.9	38	2.1	
Mastectomy	1071	982	91.7	89	8.3	

Number of patients in total and number and percentage of patients with LN metastases is given for each value of patient or tumour characteristics. The difference in LN metastases between the values was statistically tested

**Table 2:** Risk factors for lymph node metastasis in the model development cohort

	Multivariable^$^ logistic regression analysis for LN metastases		
	OR	95% CI	*p*-value
Age			
Linear	0.97	0.95–0.99	<0.001
Detection mode			
Screen-detected	1		
Otherwise	1.55	1.01–2.38	0.047
Palpable			
No	1		
Yes	2.06	1.34–3.18	0.001
BI-RADS score			<0.001
3	0.72	0.36–1.43	0.346
4	1		
5	2.41	1.53–3.78	<0.001
DCIS histological grade at biopsy			0.028
Low	1		
Intermediate	3.01	1.27–7.15	0.012
High	3.20	1.36–7.54	0.008
Suspected invasive component biopsy			
No	1		
Yes	1.86	1.01–3.41	0.045
Intercept	0.0535		

^$^Based on pooled analysis after multiple imputation of missing values; 379 of 2892 (13%) had missing data, 81 for detection mode, 148 for palpability, 223 for BI-RADS score and 84 for DCIS grade

The odds ratio (OR) and 95% confidence interval (CI) are given for univariable and multivariable logistic regression analyses for the risk of LN metastases

The interaction variable between age and biopsy grade had a *p*-value of 0.108 in multivariable analyses. Age as a linear continuous variable had a *p*-value of *p* < 0.001. Adding a quadratic term of age did not lead to a significant improvement of the model (*p* = 0.776), nor did the addition of spline variables. The effects of all other included variables were statistically significant (*p* < 0.05), and therefore selection of variables was not needed. Based on these multivariable analyses, a prediction model was constructed using the following variables: age as a continuous variable, detection mode, palpability, BI-RADS score, DCIS grade and presence of a suspected invasive component at biopsy. The predicted risk according to the model is given by:

$${\text{Predicted}} \,\,{\text{risk }} = \left( {\frac{1}{1 + \exp \left( \eta \right) }} \right) \times 100\% , \,\,\,{\text{and}}$$

$$\begin{aligned} \eta = & \, - \,2.9282 - 0.0318 \times {\text{age}} + 0.4367 \times \left( {{\text{Detection}} \,\,{\text{mode}} = {\text{otherwise}}} \right) \\ & + \,0.7231 \times \left( {{\text{Palpable}} = {\text{True}}} \right) - 0.3291 \times \left( {{\text{BIRADS}} = 3} \right) + 0.8786 \times \left( {{\text{BIRADS}} = 5} \right) \\ & + \,1.1032 \times \left( {{\text{DCIS }}\,\,{\text{grade}} = {\text{intermediate}}} \right) + 1.1625 \times \left( {{\text{DCIS}}\, {\text{grade}} = {\text{high}}} \right) \\ & + \,0.6198 \times \left( {{\text{Suspected}}\,\, {\text{Invasive}}\,{\text{Component}} = {\text{True}}} \right) \\ \end{aligned}$$

With this equation, the risk of having axillary LN metastasis was calculated for each of the 2892 DCIS patients. Supplement 4 provides some arbitrarily chosen examples of the predicted risks. The predicted risk of an individual patient can be calculated in a nomogram available on the prediction model platform Evidencio (https://www.evidencio.com/models/show/1858).

The mean predicted risk was 4.4%, with range 0.4–40.4% and percentiles 10: 1.2%, 25: 2.0%, 50: 2.8%, 75: 5.0% and 90: 9.7%. In total, for 24% of patients with DCIS (*n* = 708) the risk of LN metastasis was above 5% and for 10% (*n* = 276) the predicted risk was above 10%. Of 1821 patients undergoing breast-conserving surgery, 16% (*n* = 300) had a risk above 5%, and of 1071 patients undergoing mastectomy, 38% (*n* = 408) had a risk above 5%. Of 127 cases with metastasis, 56 had a predicted risk of less than 5% and 71 had a predicted risk above 5%. We compared the predicted risk of metastases with the predicted risk of upstaging to invasive breast cancer at surgery using the model we developed in previous research.^5^ The highest percentile group of risk of invasive breast cancer had a mean predicted risk of metastases of 10% (see Supplement 5).

### Model Performance

**Fig. 1:**
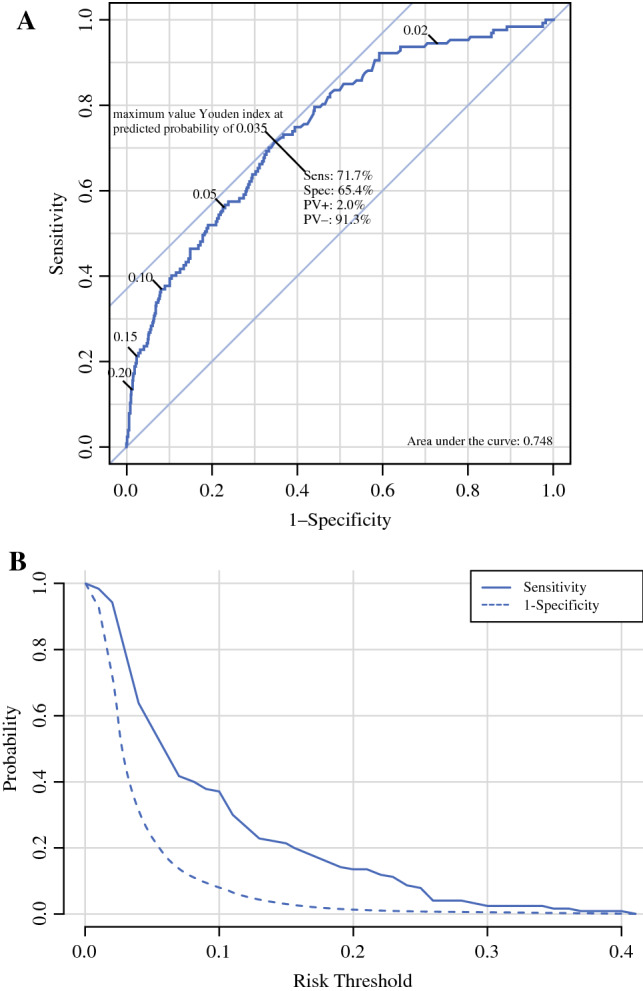
**A** Receiver operating characteristic (ROC) curve for discrimination of the prediction model. **B** The sensitivity and 1-specificity for probability thresholds. The graphs shows the sensitivity and specificity for probability thresholds of the prediction model; for instance at a predicted risk for LN metastases of 3%, the sensitivity was 0.80 and the specificity was 0.56. For a predicted risk of 5%, these were 0.56 and 0.77, and for a predicted risk of 10%, these were 0.37 and 0.92

**Fig. 2:**
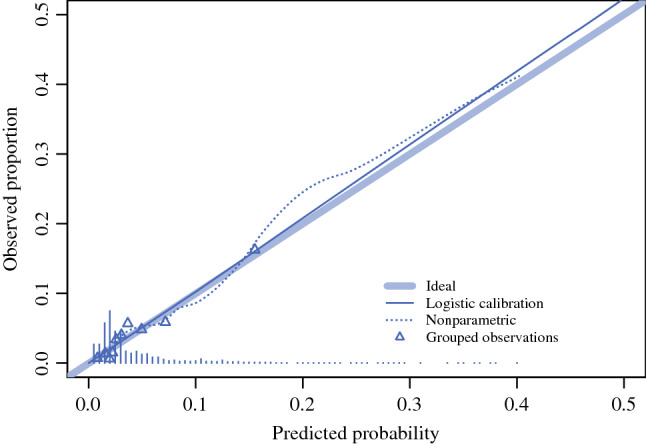
Calibration plot of the prediction model in the model development cohort. The calibration curves represent the observed versus the predicted probabilities. The ideal line corresponds to a perfect calibration (i.e., observed and predicted probabilities overlapping). A logistic and nonparametric calibration of the probabilities was fit on the observations. Triangles represent patients grouped based on similar predicted risk, and each triangle represents ten percent of the patients. The distribution of predicted risks is indicated with spikes at the bottom of the graph

The ability of the model to predict metastases is shown with the ROC curve (Fig. 1a), the threshold curve (Fig. 1b) and the calibration plot (Fig. 2). In these figures, the sensitivity shows the rate of patients with metastasis that were correctly predicted as high-risk, and the specificity shows the rate of cases with no metastasis that were correctly predicted as low-risk. At a risk threshold of 2%, the sensitivity of the model was 95% and the specificity was 27%, and at a risk threshold of 10% the sensitivity of the model was 37% and the specificity was 92%. The ROC curve had an AUC (c-index) of 0.745, which rose to 0.748 after correction for optimism by bootstrapping. The calibration plot shows the observed rate of metastasis as a function of the predicted rate. The calibration plot had a slope of 1.029 and an intercept of 0.090.

**Fig. 3:**
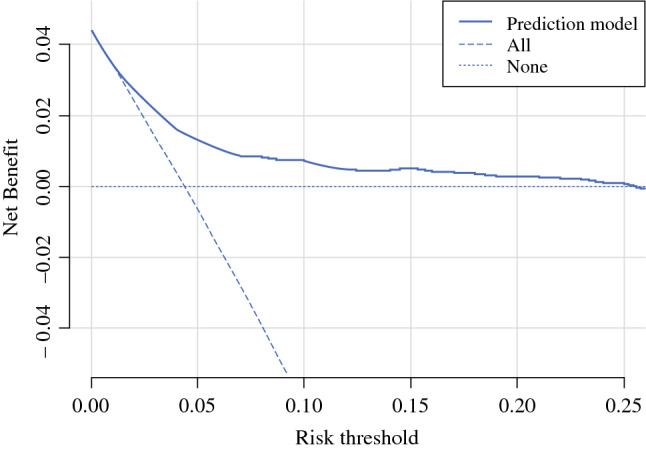
Decision Curve Analysis for risk of lymph node metastasis in the model development cohort. The net benefit of the prediction model was plotted versus the threshold probability (i.e., cut-off). In the decision curve analysis, the net benefit is also given, assuming that axillary staging is performed for none of the patients and for all patients. The prediction model has clinical utility at thresholds above the none curve and on the right of the all curve

The potential utility of the model in clinical practice is shown in Fig. 3, which presents the decision curve analysis. At a risk threshold of 2% there were 120 true positives and 2014 false positives, resulting in a net benefit 0.027. At a risk threshold of 10% there were 47 true positives and 229 false positives, resulting in a net benefit 0.007. The net benefit of the model was higher than assuming that no patient had metastases for risk thresholds up to 25%. In this dataset, 99% of patients had a risk of at most 25%.

### Model Validation

**Fig. 4:**
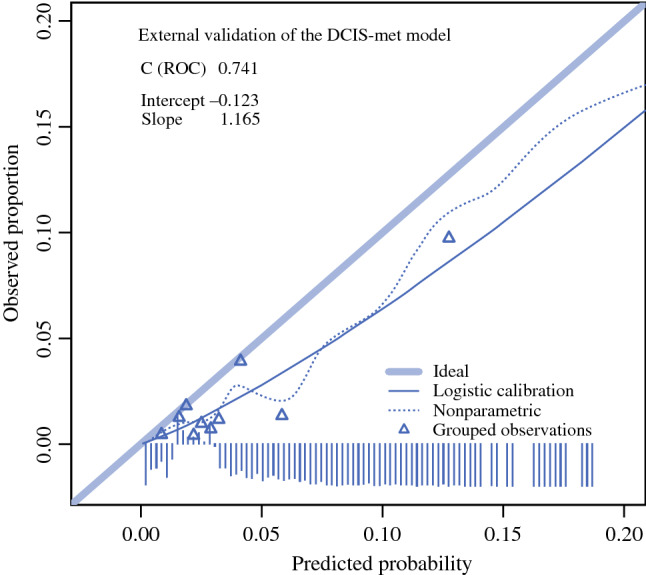
Calibration plot of the prediction model in the model validation cohort. The calibration curves represent the observed versus the predicted probabilities. The deviation of the grouped observation from the ideal line shows the proportion of risk overestimation of the model per grouped observation

**Fig. 5:**
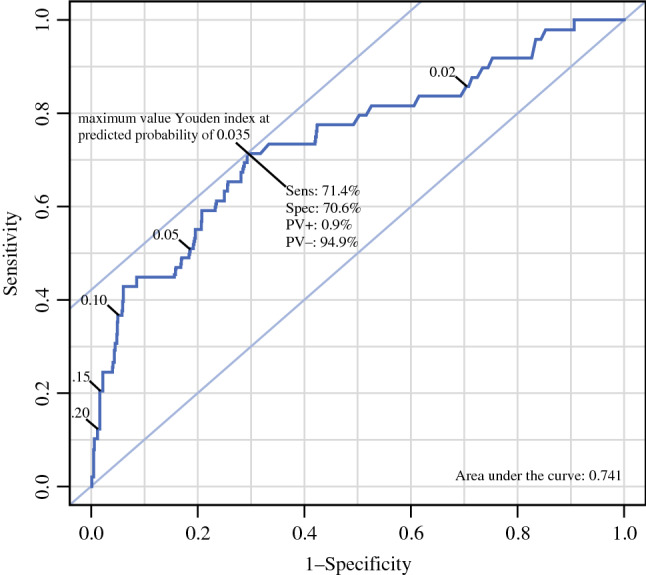
Receiver operating characteristic (ROC) curve of the model validation cohort. At each probability threshold of the model, the sensitivity and specificity are given. Some thresholds are given in the graph. The Youden’s index is calculated as follows: sensitivity + specificity − 1, for each threshold probability. At the maximum, the combination of sensitivity and specificity is optimal. At the maximum value of the Youden’s index, the sensitivity, the specificity, the positive predictive value and the negative predictive value are calculated

The calibration plot shows divergence of the cohort data from the model data, as the observed proportions are lower than the predicted proportions (see Fig. 4). The AUC in the validation cohort was 0.741, with a 95% confidence interval of 0.662–0.820 (see Fig. 5). The maximum value of Youden’s index was found at a predicted risk of 3.5%, with a sensitivity of 71.4% and a specificity of 70.6%.

## Discussion

The aim of this study was to develop a prediction model for LN metastases after a biopsy-proven DCIS to support clinical decision-making and to prevent redundant axillary staging. The study was based on two cohorts: a development cohort of 2892 patients and a validation cohort of 2296 patients. The model included the previously identified risk factors age, detection mode, palpability, BI-RADS score, DCIS grade and presence of a histologically suspected invasive component at biopsy. Risks of individual patients can be calculated at https://www.evidencio.com/models/show/1858.

For SLNB and ALND combined, we found a metastasis rate of 4.4% in 2892 patients that underwent SLNB or ALND in combination with an upstaging rate of 20% in the model development cohort and a metastasis rate of 2.2% and an upstaging rate of 18% in the validation cohort. The differences between our two cohorts might be due to a combination of factors. For instance, differences in diagnostic work-up to find invasive cancers preoperatively, resulting in more biopsy specimens, and therefore differences in upstaging rates and thus indirectly also in metastases. Also, differences in metastasis rate between cohorts can be due to differences in diagnostic work-up to find axillary metastases preoperatively and at secondary SLNB or ALND. The metastasis rate of our validation cohort is comparable with the rates of other studies; the studies of Prendeville et al., Heymans et al. and van Roozendaal et al., with 294, 240 and 910 patients, respectively, found rates of < 1%, 2.1% and 2.9%, respectively.^3,4,13^ These studies reported upstaging rates of 18%, 19% and 17%, respectively.

Some of the risk factors for LN metastasis which were taken into account in our DCIS-met model were also reported by previous studies as influencing the risk on LN metastasis. In the study by Francis, age, palpability and suspicion of microinvasion were significant, but only in univariable analysis.^1^ In multivariable analysis, the remaining significant risk factors were the biopsy method, a papillary histological subtype, DCIS size of > 2 cm and number of interventions (biopsies and surgeries). In univariable analysis, Xiao et al.^14^ found that metastases were associated with tumour size and grade. Size could not be evaluated in this study since the registries used to build the cohorts do not register the extent of the DCIS. For larger DCIS a mastectomy might more often be indicated than breast-conserving surgery. Since we found a metastasis rate for breast-conserving surgery of 2% and a metastasis rate for mastectomy of 8%, we might speculate that size would have been a risk factor in the cohorts of this study too.

The model we developed had an AUC of 0.74. In model development, this is considered a model with a fair discriminative ability. Uemoto reported an AUC of 0.67 for size as risk factor in their study of 277 patients, and Kim et al. made a prediction model for metastasis with an AUC of 0.746 based on palpability and on DCIS grade and size in a study of 506 patients.^10^ Our validation cohort comprised patients with recent incidences of DCIS. In this cohort, the average rate of metastasis was lower, and therefore also the absolute risks for each of the risk factors. Thus, the DCIS-met model gave an overestimation of the risk of metastasis in the validation cohort. Even in the validation cohort, patients can be identified with an increased relative risk of metastasis. The true probability of metastasis may differ among countries and cohorts, which may lead to larger deviations between the predicted and observed probabilities for some cohorts. Therefore, it would be advisable to compare the cohort in which the model will be used to the development cohort of study.

The opinions about the use of SLNB are changing. Initially, the fact that SLNB had a lower complication rate than full axillary staging led to a more frequent use of SLNB.^15^ Recent studies, however, advised not to perform SLNB routinely,^1,16^ or not to perform SLNB at all^3,4^ because of the low rate of metastasis. Also, the SLNB procedure has its own risk of complications.^17^ In addition, others stated that a positive lymph node hardly changes the selection of patients for adjuvant treatment.^4^ However, in our data, 235 patients with invasive breast cancer had an indication for adjuvant chemotherapy (39% of the upstaged patients), and we found that 42 of these patients would not have had an indication for adjuvant chemotherapy without axillary staging since the indication was based only on the finding of a positive lymph node. These 42 patients were 7% of patients with invasive breast cancer. Also, positive lymph nodes might change the decision on adjuvant radiotherapy. Therefore we think the SLNB is still of value for some patients with DCIS. Also, current treatment guidelines still recommend considering SLNB. For example, in the UK, for mastectomy for all patients, and for breast-conserving surgery if patients are at high risk of invasive disease.^6^ In the US, for mastectomy and for breast-conserving surgery if the excision is in an anatomic location compromising the performance of a future SLN procedure.^18^ In the Netherlands at the time of the development cohort it was recommended to consider SLNB for mastectomy and in case of breast-conserving surgery if some specific risk factors were present.^19^ The current Dutch guideline states that the SLNB can be considered in case of risk factors, irrespectively of the type of surgery.^7^ However, most current guidelines still make a distinction in patients undergoing breast-conserving surgery and patients undergoing mastectomy in the use of SLNB for biopsy-proven DCIS. Selecting patients based on risk factors may blur this distinction. For guidelines that do not recommend SLNB in patients undergoing breast-conserving surgery, selecting patients based on the prediction model may lead to SLNB in selected patients. On the other hand, for guidelines recommending SLNB in patients undergoing mastectomy, the prediction model may lead to omitting SLNB in selected cases.

A strength of this study was that the model was developed and validated on large patient cohorts based on routine clinical practice. These cohorts are from a country with a National Breast Cancer Screening Programme, which resulted in high incidences of DCIS and affected the patient and tumour characteristics. Information on palpability was available. This might be a referral reason for patients detected outside the screening program. Other referral reasons were unknown. In the development cohort, 530 patients were not detected in the National Breast Screening Programme and the DCIS was not palpable, 19 of these (3.6%) had metastases. A limited number of 32 patients with pure DCIS underwent a secondary axillary dissection, for unknown reasons. Some patients were excluded during model development, and therefore the model cannot be used for patients with previous ipsilateral DCIS or invasive breast cancer, patients with biopsy-proven micro-invasive cancer, nor for patients who underwent excisional biopsy. Of the selected patients, 13% in the model development cohort and 5% in the validation cohort had missing data for one or more risk factors. However, it is reassuring that all AUCs are almost identical: the uncorrected AUC of 0.745, the optimism-corrected AUC of 0.748, and the validation AUC of 0.741. At the maximum value of Youden’s index, the sensitivity was 71.4%. The decision curve analysis showed that the model was of benefit to 99% of the patients in the model development cohort since the model is valid for patients with a predicted risk below 25%. In the decision curve analysis, it was shown that the net benefit, in which the true positive and false positive predictions are weighed against each other, is higher than assuming all patients have metastases, and also higher than assuming no patient has a metastasis. Therefore, it is clinically useful to use the model for selecting patients for SLNB.

In conclusion, the DCIS-met model is based on factors available in daily clinical practice, and this model can support clinical decisions on axillary staging in patients with biopsy-proven DCIS.

## Supplementary Information

Below is the link to the electronic supplementary material.

Supplementary file1 (PDF 186 KB)

Supplementary file2 (PDF 159 KB)

Supplementary file3 (PDF 189 KB)

Supplementary file4 (PDF 155 KB)

Supplementary file5 (PDF 167 KB)
