# Supplementary material for: Predicting Lymph Node Metastases in Patients with Biopsy-Proven Ductal Carcinoma In Situ of the Breast: Development and Validation of the DCIS-met Model
Source: Ann Surg Oncol. 2022 Dec 10;30(4):2142–51. doi: 10.1245/s10434-022-12900-7 (PMC10027636; doi:10.1245/s10434-022-12900-7)
Supplement: Supplementary file 1 — Supplementary file1 (PDF 186 KB) [file 10434_2022_12900_MOESM1_ESM.pdf]

Supplement 1:
Axillary staging in combination with the diagnosis at excision in the model development cohort

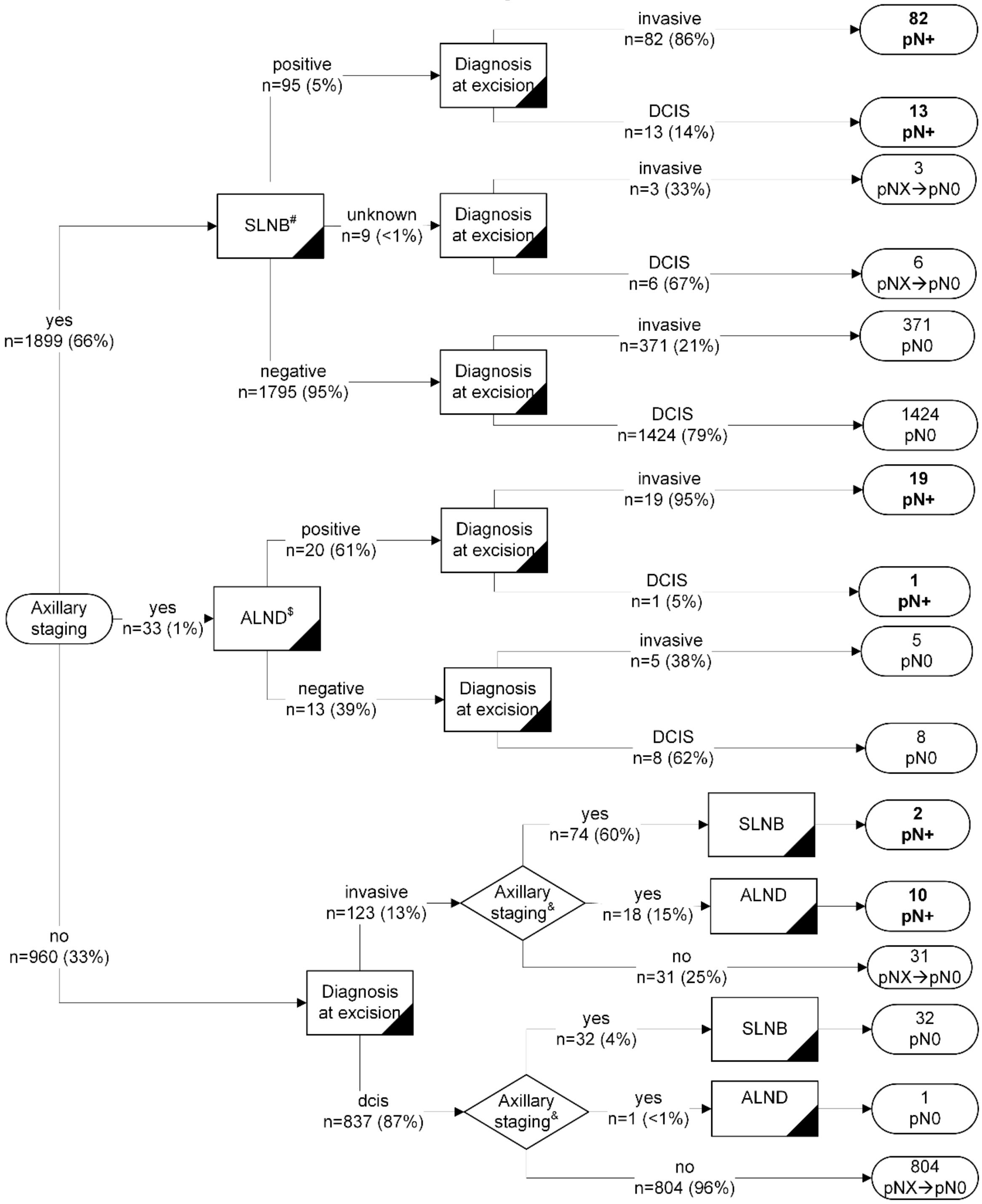

Axillary staging for the 2892 patients of the development cohort, given as a function of the following: as primary or secondary staging, staging by SLNB or ALND, and by diagnosis DCIS or invasive breast cancer at excision. The result of the staging is given as pN status for each arm of the flow chart; in a total 127 patients pN1. In the model development pNX is considered pN0.
The SLNB was performed before or at the first excision
$ The ALND was performed at the first excision (this can be preceded by an SLND)
& The SLNB or ALND after the first resection, during second resection or as stand-alone procedure
