# Supplementary material for: Predicting Lymph Node Metastases in Patients with Biopsy-Proven Ductal Carcinoma In Situ of the Breast: Development and Validation of the DCIS-met Model
Source: Ann Surg Oncol. 2022 Dec 10;30(4):2142–51. doi: 10.1245/s10434-022-12900-7 (PMC10027636; doi:10.1245/s10434-022-12900-7)
Supplement: Supplementary file 2 — Supplementary file2 (PDF 159 KB) [file 10434_2022_12900_MOESM2_ESM.pdf]

Supplement 2:
Characteristics of invasive breast cancer cases with lymph node metastasis after biopsy-proven DCIS in the model development cohort

Tumour characteristics				N	%
Morphology				113	
	Lobular			2	2
	Ductal			105	93
	Mixed Ductal and Lobular			3	3
	Other			3	3
Grade of the invasive tumour				103	
	I			13	13
	II			40	39
	III			50	48
ER receptor				109	
	Negative			23	21
	Positive			86	79
PR receptor				109	
	Negative			44	40
	Positive			65	60
Her2Neu				109	
	Negative			76	70
	Positive			33	30
Receptor combinations				106	
	ER-	PR-	Her2Neu -	8	8
	ER+		Her2Neu -	66	62
	ER-	PR-	Her2Neu +	14	13
	ER+		Her2Neu +	17	16
	ER-	PR+	Her2Neu +	1	<1
Tumour size (in mm)				109	
	mean - median (range)			16.5 - 11 (1 - 90)	
TNM stage				113	
	I A			0	0
	I B			16	14
	II A			52	46
	II B			20	18
	III A			16	14
	III B			0	0
	III C			9	8
	IV			0	0
