# Supplementary material for: Predicting Lymph Node Metastases in Patients with Biopsy-Proven Ductal Carcinoma In Situ of the Breast: Development and Validation of the DCIS-met Model
Source: Ann Surg Oncol. 2022 Dec 10;30(4):2142–51. doi: 10.1245/s10434-022-12900-7 (PMC10027636; doi:10.1245/s10434-022-12900-7)
Supplement: Supplementary file 3 — Supplementary file3 (PDF 189 KB) [file 10434_2022_12900_MOESM3_ESM.pdf]

Supplement 3:
Risk of lymph node metastases in primary or secondary axillary evaluation in the model validation cohort

	Biopsies with DCIS	Lymph Node metastasis				
		No		Yes		
		N	%	N	%	p-value
Total	2269	2220	98	49	2.2	
Age (mean, median, range)	59.4, 59 (23-90)	59.5, 59 (23-90)		56.0, 57 (27-78)		0.055*
Detection mode						<0.001&
Screening	1530	1509	98.6	21	1.4	
Otherwise	739	711	96.2	28	3.8	
Palpable						<0.001&
No	1894	1867	98.6	27	1.4	
Yes	375	353	5.9	22	5.9	
DCIS grade						0.01#
Low	263	263	100	0	0	
Intermediate	954	932	97.7	22	2.3	
High	1052	1025	97.4	27	2.6	
BI-RADS score						<0.001#
3	117	116	99.2	1	0.9	
4	1939	1907	98.4	32	1.7	
5	213	197	92.5	16	7.5	
Suspected invasive component at biopsy						0.003#
No	2221	2177	98.0	44	2.0	
Yes	48	43	89.6	5	10.4	

The p-value was determined with *Mann-Whitney U test, # Fisher's exact test, & Chi-square test
