# Supplementary material for: Predicting Lymph Node Metastases in Patients with Biopsy-Proven Ductal Carcinoma In Situ of the Breast: Development and Validation of the DCIS-met Model
Source: Ann Surg Oncol. 2022 Dec 10;30(4):2142–51. doi: 10.1245/s10434-022-12900-7 (PMC10027636; doi:10.1245/s10434-022-12900-7)
Supplement: Supplementary file 4 — Supplementary file4 (PDF 155 KB) [file 10434_2022_12900_MOESM4_ESM.pdf]

Supplement 4:
Predicted risk of LN metastasis for example cases in the model development cohort

Preoperative characteristics	Example case									
	**1**	**2**	**3**	**4**	**5**	**6**	**7**	**8**	**9**	**10**
Age	50	50	64	74	50	64	64	64	45	45
Detection mode	Screening	Screening	Screening	Screening	Screening	Otherwise	Otherwise	Otherwise	Otherwise	Otherwise
Palpable	No	No	No	No	No	Yes	Yes	Yes	Yes	Yes
BI-RADS score	4	4	4	4	5	4	5	5	4	5
DCIS histological grade at biopsy$	Low	High	High	High	High	Interm.	High	High	Interm.	High
Suspected invasive component biopsy	No	No	No	No	No	No	No	Yes	No	No
Predicted risk (%)	1.1	3.4	2.2	1.6	7.7	6.3	14.6	24.2	10.9	23.9
$ Intermediate is given as Interm..										
