# Supplementary material for: Predicting Lymph Node Metastases in Patients with Biopsy-Proven Ductal Carcinoma In Situ of the Breast: Development and Validation of the DCIS-met Model
Source: Ann Surg Oncol. 2022 Dec 10;30(4):2142–51. doi: 10.1245/s10434-022-12900-7 (PMC10027636; doi:10.1245/s10434-022-12900-7)
Supplement: Supplementary file 5 — Supplementary file5 (PDF 167 KB) [file 10434_2022_12900_MOESM5_ESM.pdf]

Supplement 5:

Risk of LN metastasis according to percentile groups of risk for upstaging to invasive breast cancer at surgery in the model development cohort

	**<20th percentile**	**20-<40th percentile**	**40 - <60th percentile**	**60 - <80th percentile**	**≥80th percentile**
Number of biopsy-proven DCIS	472	526	643	632	619
Mean predicted risk of upstaging to invasive cancer#	11.6%	14.2%	14.8 %	21.9%	39.1%
Mean predicted risk of metastasis	1.2%	2.4%	2.6%	4.4%	10.2%
Observed rate of metastasis	0.9%	2.7%	2.2%	4.3%	11.0%

In previous research, we developed a model for the risk of invasive breast cancer, diagnosed at excision after a DCIS diagnosis at biopsy.(5) Patients were divided into risk groups based on that model.

For each risk group, the risk of lymph node metastasis was calculated with the prediction model developed in this study.

Also, the observed rate of metastasis in for each of these risk groups is given.
